# Enzyme replacement therapy and fatigue in adults with Pompe disease

**DOI:** 10.1186/1471-2474-14-S2-P16

**Published:** 2013-05-29

**Authors:** Deniz Güngör, Juna de Vries, Esther Brusse, Michelle Kruijshaar, Linda van den Berg, Wim Hop, Arnold Reuser, Pieter van Doorn, Marloes Hagemans, Iris Plug, Ans van der Ploeg

**Affiliations:** 1 Departments of Pediatrics, Neurology and Clinical Genetics, Center for Lysosomal and Metabolic Diseases, Erasmus MC University Medical Center, Rotterdam, The Netherlands

## Introduction

Fatigue is a common and often disabling symptom among both mildly and severely affected adult patients with Pompe disease. Our objective was to determine whether enzyme replacement therapy (ERT) reduces fatigue in adult patients with Pompe disease.

## Methods

Data was collected as part of an international longitudinal survey (‘IPA/ Erasmus MC Pompe Survey’). Fatigue was measured with the Fatigue Severity Scale (FSS). Repeated measurements ANOVA were used to analyze the data over time. We also evaluated muscle strength using the Medical Research Council (MRC) scale, measured pulmonary function as Forced Vital Capacity (FVC), and assessed depression using the Hospital Anxiety and Depression scale (HADS).

## Results

We followed 163 patients for a median of 4 years before ERT and for 3 years during ERT. At start of ERT, 68% of patients were severely fatigued (FSS ≥ 5), against 55% at their last follow-up. During ERT, mean FSS scores declined significantly (-0.13 score points per year; p<0.001) – a significant improvement on the period before treatment (difference -0.14 FSS score-points per year, p <0.01). Patients’ improvements in fatigue were correlated with their response to ERT in muscle strength (correlation coefficient: -0.55) and depression (correlation coefficient: 0.34), but not with the effect of ERT on pulmonary function.

## Conclusion

Fatigue is a prominent symptom in adult Pompe disease. Although it decreased during ERT, over half of the patients remained severely fatigued after a median of 3 years of therapy. To manage fatigue optimally in these patients, ERT should therefore be combined with other strategies that target fatigue. Further investigation is needed into the exact role of muscle strength, depression and other factors that may be associated with fatigue.

